# Experimental Study on OC PEMFC Performance Improvement and MEA Parameter Optimization Under Water Shortage Conditions

**DOI:** 10.3390/membranes15120356

**Published:** 2025-11-26

**Authors:** Jianan Wang, Di Tang, Tianshu Liao, Xiangqian Zhang, Feng Cheng, Lingfeng Gao

**Affiliations:** 1 Wuhan Institute of Marine Electric Propulsion, Wuhan 430000, China; 18371996724@163.com; 2 Wuhan Institute of Hydrogen and Fuel Cell Industrial Technology, Wuhan 430000, China; 15927087254@163.com (D.T.); lts961217@126.com (T.L.); 18371996724@139.com (X.Z.); joy19960717@126.com (F.C.)

**Keywords:** open-cathode proton exchange membrane fuel cell, membrane electrode assembly, air-cooled, electrochemical impedance spectroscopy

## Abstract

Optimizing the MEA structure is crucial for enhancing the performance of open-cathode PEMFCs under water shortage conditions. By investigating the impact of gradient ambient temperature on performance, it is highlighted that cathode catalyst layer hydration deeply affects proton conduction in the membrane and three-phase boundary formation. These issues consequently increase ohmic resistance and cathode activation resistance as seen via polarization curve comparison and the electrochemical impedance spectroscopy analysis method, ultimately degrading overall stack voltage output under the same current density. Under indoor temperature and humidity conditions, an orthogonal experiment was designed to validate the sensitivity analysis on the cathode I/C ratio (0.74–0.9) and catalyst layer thickness (8, 12 μm) by controlling the catalyst-coated membrane manufacture process; GDL thickness (185–324 μm) and pore structure were also investigated, combining parameter characterization techniques like MIP and BET. It is shown that with an I/C ratio of 0.86, a medium GDL pore structure and a higher catalyst layer thickness of 12 μm bring better performance output, especially when the OC PEMFC is 700 mA/cm^2^ @ 0.62 V.

## 1. Introduction

Open-cathode proton exchange membrane fuel cells have demonstrated distinct advantages in drone power supplies and portable generating facilities, thanks to their simplified system structure, low maintenance requirements, and rapid start-up capabilities [1,2]. In OC PEMFCs, the cathode channel is exposed to ambient air, allowing direct atmospheric intake to supply the cathode reaction gas. The resulting forced air convection simultaneously provides stack cooling. This design eliminates the complex water circulation pipeline required in the water-cooled PEMFC (WC PEMFC) system, significantly reducing the parasitic power losses while enhancing overall power density [3].

However, this thermal management approach creates challenging operating conditions for the membrane electrode assembly [4]. Elevated ambient temperatures accelerate MEA water dehydration. When the air flow supply is increased for more heat dissipation, more water is removed from the MEA. In addition, at normal ambient temperature, the water content in the air is not sufficient for PEMFC. This triple effect critically impacts cell performance: water loss not only diminishes the proton exchange membrane’s (PEM) conductivity but also impairs oxygen dissolution at the cathode’s three-phase boundaries. Therefore, enhancing OC PEMFC performance requires focusing on optimizing water retention in the MEA under water shortage conditions [5].

Structurally, optimizing the design of end plates [6] and flow channels [2,3,7] can achieve lower contact resistance and improved gas transport in OC PEMFCs. Performance can also be enhanced by control strategies like anode purging strategies [8] and cathode intake fan control [9]. Furthermore, water and heat management levels at the MEA are particularly critical [10]. Unlike WC PEMFCs that face flooding issues [11,12], OC PEMFCs, affected by air-cooling conditions, often operate in a water-deficient state. Thus, it is important to investigate the water-retaining mechanism and optimization like GDL [13] and CL [10,14]. The current technological challenges in OC PEMFC development include the following: Firstly, single-factor optimization approaches are insufficient to address the complex water–heat–mass coupling issues. Secondly, most OC PEMFC MEA parameters are inherited from WC PEMFC MEA designs [15,16,17,18], which require specialized parameter sensitivity analysis to enable targeted performance enhancement.

Presently, with the rapid growth of portable power sources and drone energy supply, new requirements have been put forward for the performance improvement and sensitivity analysis of OC PEMFCs. In 2024, an experimental design of high-performance OC PEMFCs [13] from the perspective of resin selection creatively proved that the resin type and EW value have an impact on the water retention inside the CL for OC PEMFCs. The current density was improved from 0.15 A/cm^2^ to 0.3 A/cm^2^ @ 0.6 V by changing the resin from EW 1100 to EW 720, verifying the importance and necessity of optimizing the MEA structure and parameters.

This research conducted systematic experiments of ambient temperature, CL I/C ratio, CL structure, and GDL structure on OC PEMFC performance. Meanwhile, in situ electrochemical impedance spectroscopy (EIS) and ex situ characterization techniques were employed to elucidate the critical role of cathode water management in OC PEMFCs. The study establishes quantitative relationships among structural parameters, water retention mechanisms, and performance optimization, enabling performance improvement under MEA water shortage conditions.

## 2. Materials and Methods

### 2.1. Materials and Preparation

**Figure 1:**
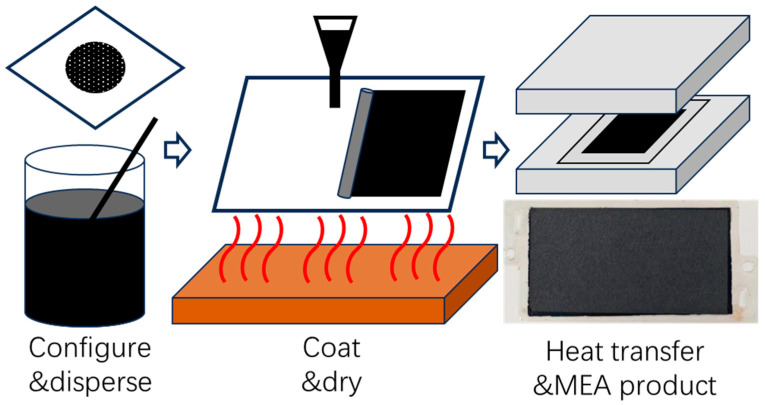
Process of MEA fabrication.

MEAs in this study are fabricated by a transfer printing process. First, a catalyst ink slurry is prepared by mixing catalyst particles, ionomer resin, and solvent in precise proportions. This homogeneous slurry is then uniformly coated to form the CL. After the drying process, thermal printing will transfer the CL onto both the cathode and anode sides of a PEM, creating a three-layer catalyst-coated membrane structure. Finally, the assembly is completed by integrating GDL and sealing gaskets to produce the seven-layer MEA. The complete fabrication process is illustrated in Figure 1: first, components are mixed and dispersed in a certain proportion, then coated and dried, and finally subjected to hot press transfer printing to produce a seven-layer MEA (including PEM, CLs, gaskets and GDLs, both anodes and cathodes).

**Figure 2:**
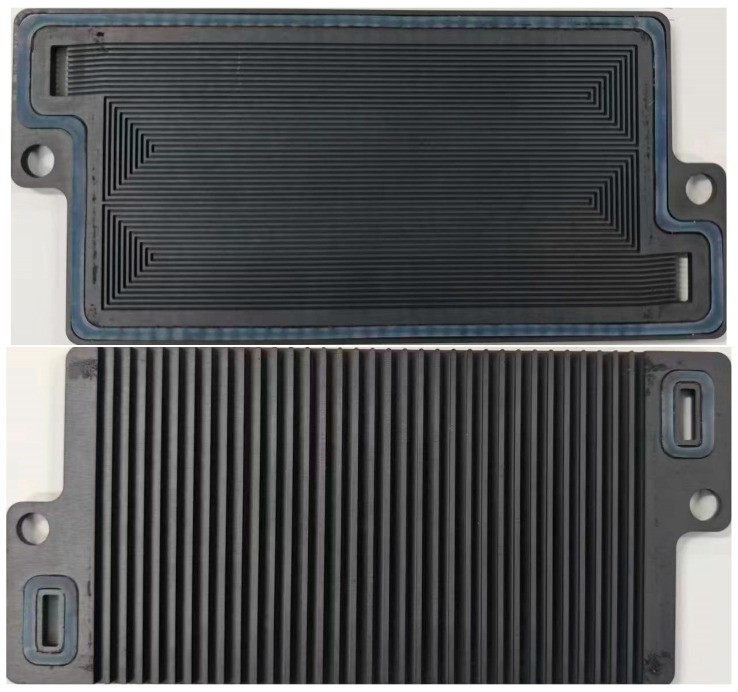
Bipolar plate used in this paper including anode channel and cathode, but no cooling water in OC PEMFCs.

The materials involved are shown in Table 1. In addition, the overall OC PEMFC includes a bipolar plate in Figure 2, and components involved in the fuel cell stack are shown in Table 2.

### 2.2. Equipment and Procedures

This study employed a cathode-open and anode dead-end pulse-emission configuration. Additionally, 99.999% purity hydrogen was supplied directly from gas cylinders without external humidification, while ambient air served as the oxidant at environmental temperature and humidity conditions. A 500 W air-cooled test station offered an anode purging protocol consisting of periodic 0.2 s openings followed by 25 s closure intervals. Thermocouples were placed in both the air inlet and air exhaust positions. A constant-speed cooling fan maintained a consistent air flow throughout the whole experiment. For electrochemical characterization, we conducted EIS measurements under galvanostatic conditions using a ZAHNER Zennium Pro from Zahner, Kronach, Germany. The frequency sweep ranged from 3000 Hz to 0.5 Hz with an AC perturbation amplitude of 5–10% of the operating current as shown in Table 3.

The CL’s surface wettability was evaluated using a sessile drop method with the following steps: Firstly, a 4 μL droplet of deionized water was deposited on the CL surface. Then, contact angle evolution was monitored for 10 s using high-speed imaging (30 fps). After the droplet of deionized water was stable on the sample, images from a high-speed camera were analyzed automatically, then distinguished by corresponding software, OneAttension standard edition. Finally, contact angles were quantified to characterize the surface hydrophobicity. The testing facility was Attension Theta Lite by Biolin Scientific from Gothenburg, Sweden. The contact angle analysis model adopted the Young–Laplace algorithm. During a single test, the high-speed camera provided hundreds of test images, and the above analysis was repeated on each image, ensuring the repeatability of the test.

The Brunauer–Emmett–Teller (BET) method was employed to analyze the CL’s micropore structure through nitrogen adsorption–desorption isotherms. Micromeritics-ASAP 2460 equipment from Malvern Panalytical in Malvern, UK, quantitatively evaluates key structural parameters including specific surface area, pore volume, and pore size distribution, enabling systematic comparison of microstructural variations across different MEA design configurations.

Mercury intrusion porosimetry (MIP) was utilized to characterize the GDL’s macropore structure. This method provides comprehensive data on pore size distribution, total pore volume, and internal surface area, allowing for detailed comparison of structural parameters between different GDL types. AutoPore 9500 equipment from Malvern Panalytical in Malvern, UK, was applied.

Det ETD-SE equipment from CIQTEK, in Hefei, China, was applied for the scanning electron microscope (SEM) test, which is necessary for CL comparison at the micron level.

**Figure 3:**
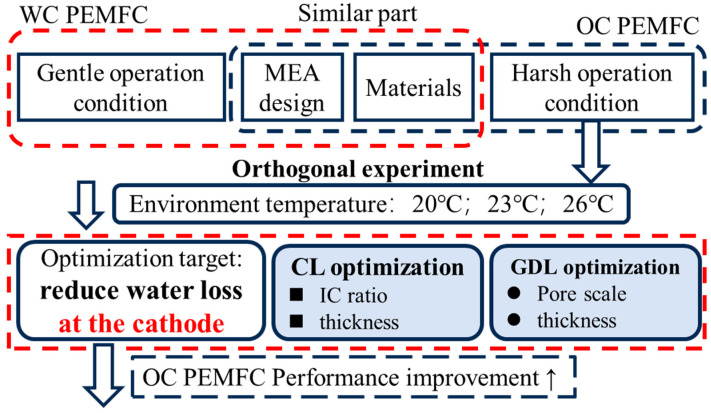
Experiment configuration and logic diagram, based on harsh operation conditions of OC PEMFCs; optimization directions and methods are fully explained.

Based on the analysis of OC PEMFC performance output, the influence of gradient ambient temperature was systematically investigated first. Polarization curves and EIS results were used for the investigation of in situ MEA conditions. Subsequently, the optimization directions of OC PEMFC were revealed: (1) cathode CL structure thickness and I/C ratio, and (2) cathode GDL structure thickness and pore scale, as shown in Figure 3. The specific orthogonal experimental design parameters are shown in Table 4.

We started conducting the OC PEMFC optimization experiment in summer 2023. At first, we did not consider the effect of changing seasons on fuel cell performance. Although our laboratory is under constant temperature and humidity conditions, the huge temperature difference between winter and summer in Wuhan, China, resulted in a temperature difference of 6 °C between indoors and outdoors. Based on this, we designed the experimental plan described in this paper and found that even a small temperature difference can bring such a large difference.

The I/C ratio is used to systematically screen WC PEMFCs. Generally speaking, an I/C ratio of 0.8 is best for WC PEMFCs under 80 °C and 100% RH. The 0.74–0.9 scope was used to determine the value of 0.8, because we had no idea which direction was correct at first.

As for the CL thickness values of 8 and 12 μm, we assumed the thicker cathode electrode pathway would lessen the possibility of water loss. By changing the MEA preparation process, such as increasing the temperature from 60 °C to 80 °C in the drying process (Figure 1), we obtained catalyst layers of different thicknesses (also different densities) under the same platinum loading conditions. Currently, we can only achieve these two gradients.

As for GDL thickness, we had the same purpose as CL thickness. Experiments for thickness selection were carried out using JNTG products, which have similar pore sizes but different thicknesses.

## 3. Results

### 3.1. Ambient Temperature Effect

This study systematically investigated the influence of ambient temperature (20 °C, 23 °C, 26 °C) on the performance of OC PEMFCs.

**Figure 4:**
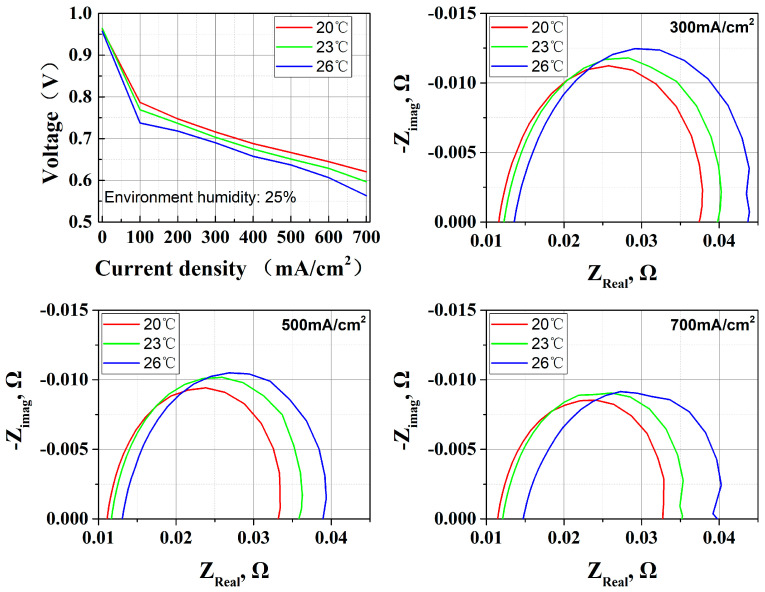
Effect of environment temperature (20 °C, 23 °C, 26 °C) on OC PEMFCs; performance and EIS analyses show that 26 °C results in both cathode polarization and ohmic resistance degradation.

The experimental data in Figure 4 demonstrates a deterioration in performance as ambient temperature escalates from 20 °C to 26 °C. While elevated temperatures theoretically enhance catalyst activity to improve electrochemical reactions, this thermal effect simultaneously accelerates MEA dehydration indirectly through intensified water evaporation. The experimental results show that the latter dominates: the proton conductivity and cathodic three-phase boundaries (TPBs) are damaged by MEA dehydration. EIS analysis corroborates this phenomenon, revealing a synchronized increase in both high-frequency impedance and intermediate-frequency impedance.

**Figure 5:**
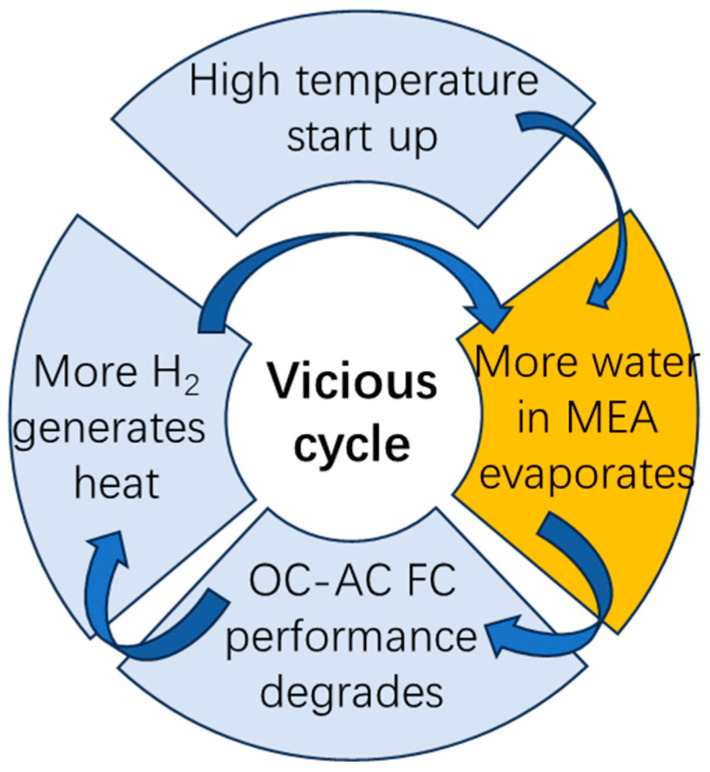
Vicious cycle of water–heat–mass coupling; high temperature leads to a series of outcomes inside the MEA.

In principle, a temperature difference of 6 °C is not enough to cause severe dehydration of the MEA. But performance degradation results demonstrate a vicious cycle effect: a high ambient temperature influences the heat cumulative effect, leading to an increase in heat dissipation difficulty. This process will increase some of the ohmic impedance, resulting in a partial decrease in voltage output and more energy being used for heating. The accumulation of heat and the decrease in heat dissipation at high temperatures work together on the MEA, causing it to lose more water compared to lower temperatures. In open-cathode air-cooling configurations, the compounding effects of environmental temperature rise and forced convection cooling paradoxically exacerbate thermal management challenges. Specifically, the system enters a detrimental feedback loop characterized by performance decline → waste heat accumulation → intensified cooling demands → accelerated component dehydration. This self-reinforcing mechanism in Figure 5 ultimately amplifies overall energy losses while weakening system stability.

The experimental findings reveal that effective water management in OC PEMFCs critically depends on cathode water retention mechanisms. Under anode dead-end configurations, water generated mainly dehydrates from the cathodic CL-GDL channel pathway. Elevated environmental temperatures exacerbate water depletion through intensified evaporation, thereby reducing PEM hydration levels and precipitating performance degradation.

### 3.2. I/C Ratio Effect

While OC PEMFCs and WC PEMFCs exhibit fundamental differences in cathode gas supply mechanisms and operational environments, they share critical commonalities in MEA structure and material composition. The I/C ratio, defined as the mass proportion between perfluorosulfonic acid ionomers and carbon in catalysts, indicates three essential functional characteristics: (1) proton conduction pathways, (2) porous structure [15], and (3) TPB formation in CL [16,17]. For OC PEMFCs, leveraging the inherent hydrophilicity of ionomers, which facilitates moisture adsorption and membrane hydration maintenance, may not only significantly enhance water retention capabilities in MEA water shortage operational scenarios but also enhance proton conductivity to improve performance thereby.

**Figure 6:**
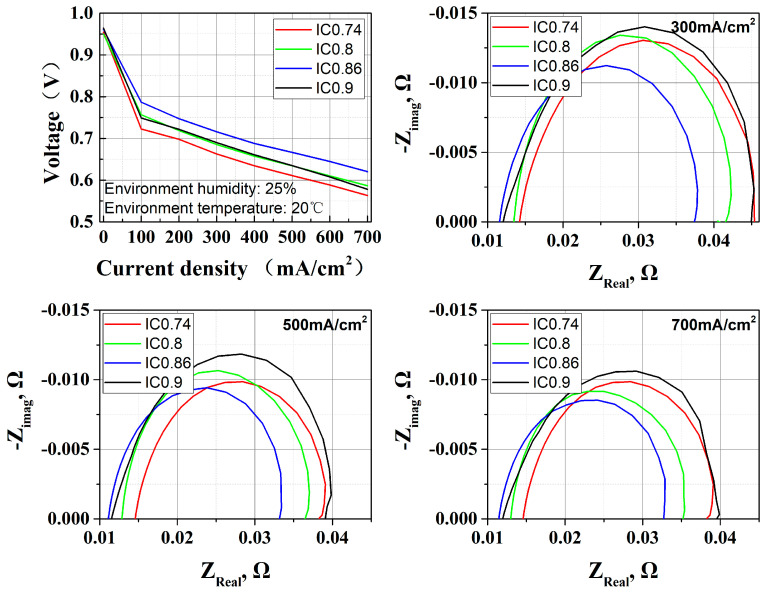
Effect of I/C ratio (0.74, 0.8, 0.86, 0.9) on OC PEMFC; performance and EIS results show that the best I/C ratio is 0.86.

This study systematically investigates the influence of the cathode CL I/C ratio on the performance of OC PEMFCs. The I/C ratios were experimentally varied at 0.74, 0.8, 0.86, and 0.9, with performance evaluation conducted through polarization curve analysis and EIS measurements at current densities of 300 mA/cm^2^, 500 mA/cm^2^, and 700 mA/cm^2^. The results in Figure 6 demonstrate that the optimal cell performance is achieved at an I/C ratio of 0.86.

**Figure 7:**
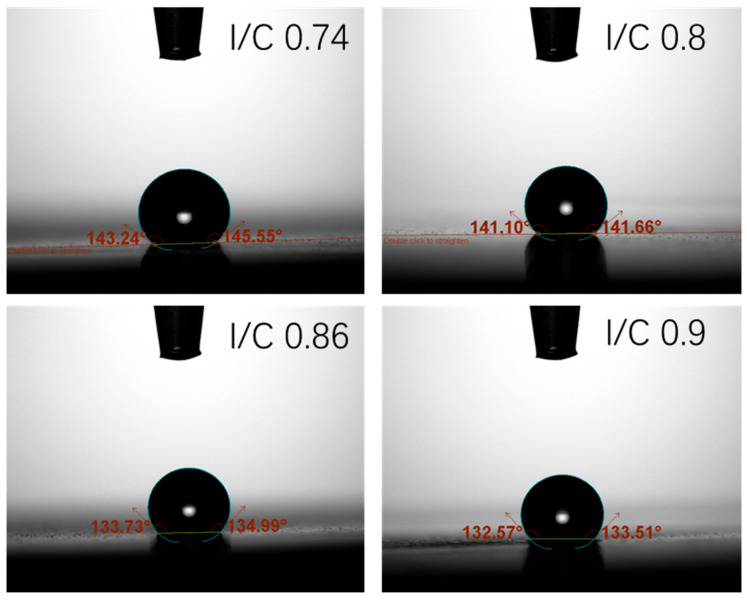
Contact angle of CL with different I/C ratios (0.74, 0.8, 0.86, 0.9).

As the cathode I/C ratio increases from 0.74 to 0.86, EIS results show (1) a reduction in ohmic impedance, and (2) a decrease in cathode activation impedance. The increased content of perfluorosulfonic acid resin in the cathode CL expands the proton conduction pathways, thereby enhancing proton conductivity and consequently reducing the ohmic impedance at the cell level. And enhanced water retention capacity of the cathode enables better moisture preservation under relatively dry operating conditions, and the contact angle in Figure 7 implies that I/C ratios of 0.86 and 0.9 lead to a more hydrophilic CL. This effect promotes the formation of additional TPBs, leading to reduced oxygen transport resistance.

However, it should be noted that when the I/C ratio was elevated from 0.86 to 0.9, the performance of the stack decreased along with the deterioration of cathode activation resistance (EIS arc section). This can be explained by two perspectives: Excessive ionomer resin will wrap the platinum–carbon catalyst, leading to the elimination of TPBs and flooding. (1) The relationship between TPB formation and I/C ratio is not linear. When the I/C ratio is above 0.9, resin gathered on the Pt/C might thicken. After the thickness exceeds the particle size of platinum, TPBs disappear. Additionally, increased hydrophilic ability in the catalyst layer can excessively adsorb liquid water, causing flooding.

### 3.3. CL Pore Structure Effect

**Figure 8:**
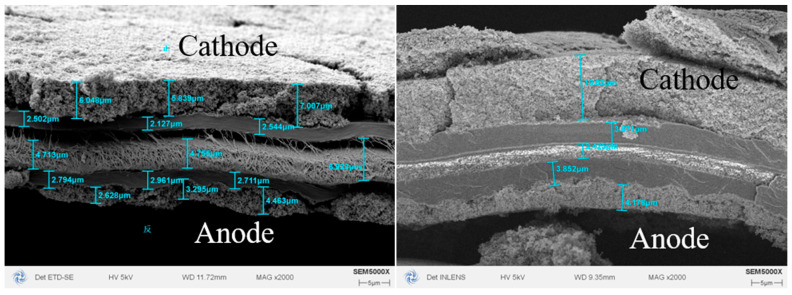
SEM images of 8 μm and 12 μm cathode CLs (on the upper side), with the same 10 μm PEM (middle) and 4 μm anode CL (in the lower side).

A porous structure in a CL offers water retention and electrochemical reaction sites. In OC PEMFCs, the optimized pore volume within CL becomes particularly crucial under indoor water content operating conditions. In this investigation, we engineered cathode CLs with controlled thicknesses of 8 μm and 12 μm while maintaining a constant platinum loading of 0.4 mg/cm^2^ through precise adjustment of the manufacturing process. Under these two CL cases, IC ratios were both 0.86, while bulk densities and porosities were passively different driven by different manufacturing parameters as shown in Figure 8.

In Figure 8, two 3-layer CCMs are shown, which have the same 10 μm PEM (middle) and 4 μm anode CL (on the lower side). On the upper side, 8 μm and 12 μm cathode CLs have been pressed to 7 μm and 10 μm. In the preparation process of CCLs above, the same procedure is used, but the difference lies in the drying process of the slurry. To manufacture catalytic layers with the same platinum loading but different catalytic layer thicknesses, different drying temperatures are used.

**Figure 9:**
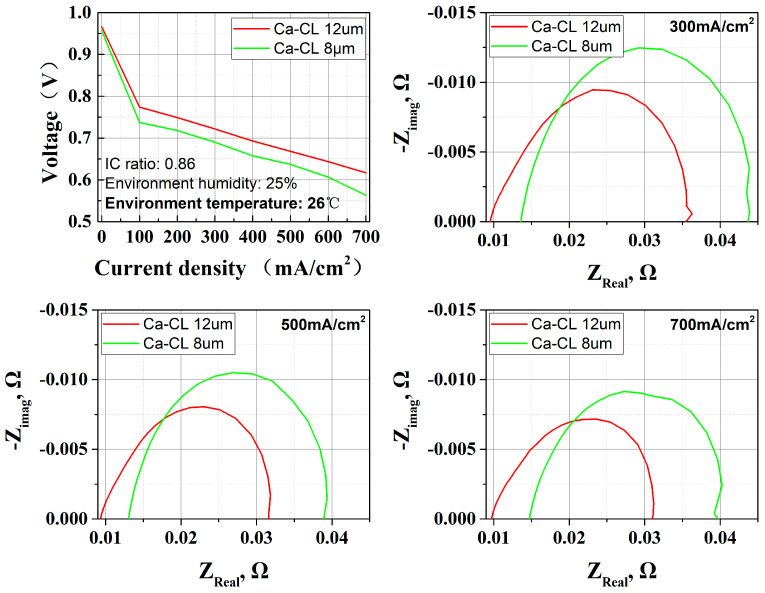
Effect of CL thickness (8 μm and 12 μm) on OC PEMFC; performance and EIS results show that 12 μm CL thickness leads to both ohmic resistance and cathode polarization resistance reduction.

BET findings demonstrate that increased CL thickness, correlating with reduced density (from 1.119 to 0.893 mg/mm^3^) and enhanced surface area (from 3.5283 m^2^/g to 4.5523 m^2^/g), induces a substantial enhancement in cell output performance. Figure 9 comprehensively illustrates these performance trends and EIS results.

The experimental results demonstrate a notable performance enhancement in thicker CLs from 8 μm to 12 μm. EIS analyses combined reveal that the enhanced performance originates from two synergistic effects facilitated by the thicker CL: (1) the more porous structure improves MEA hydration through effective water retention, thereby reducing proton transport resistance (as evidenced by ohmic impedance reduction from 0.15 Ω at 8 μm to 0.10 Ω at 12 μm in the high-frequency region); (2) the expanded TPB network promotes oxygen reduction reaction (ORR) kinetic performance, which is corroborated by the significant decrease in charge transfer impedance observed in the low-frequency region.

**Figure 10:**
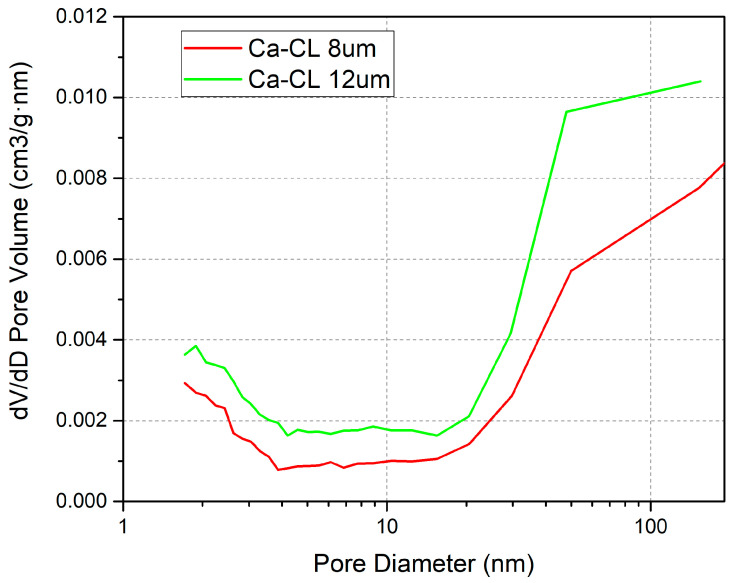
Pore distribution of CLs with 12 μm and 8 μm thickness values in BET results.

According to the BET results of the CLs, different thicknesses of CLs have the following characteristics in terms of pore structure (Figure 10).

The increase in CL thickness (decrease in density) preserves necessary moisture in pores, maintaining the hydration state of the membrane electrode.

### 3.4. GDL Structure Effect

In OC PEMFCs, water generated at the cathode CL dissipates through the GDL and is then removed by forced convection in the cathode channel. These characteristics necessitate optimization of GDL structure to achieve effective water retention. Two critical parameters of GDL are investigated in this paper: water dissipation distance (various GDL thicknesses) and water dissipation difficulty (various GDL pore structures).

**Figure 11:**
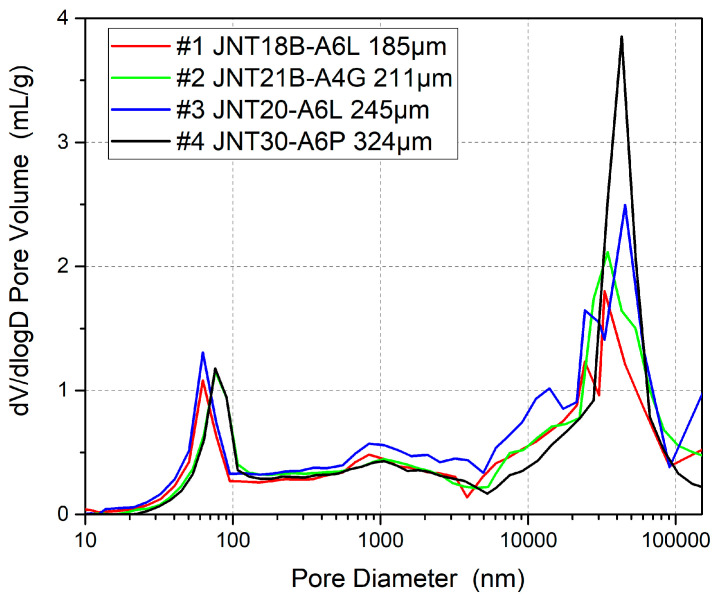
MIP results of #1–#4 GDLs, which have 4 different levels of thickness, manifesting a similar pore size distribution.

In this experiment, four different thicknesses of GDLs were selected for comparison, with thicknesses of 185 μm (#1), 211 μm (#2), 245 μm (#3), and 324 μm (#4) to investigate the effect of the water discharge distance on the performance of OC PEMFCs. GDLs #1–#4 have a similar microporous layer (MPL) pore size and volume according to pore size distribution results, and a similar MPL thickness and manufacturer, ensuring that the main variable is carbon paper thickness. As the thickness of the GDL varies, the pore volume of the carbon paper substrate changes accordingly, but the pore sizes are within a similar range. The parameters are shown in Table 1, and the pore structure is shown in Figure 11; the 10–100 nm range belongs to MPL pores, which are similar thanks to the same preparation process, and the 10,000–100,000 nm range belongs to carbon paper pores, which are consistent in scope but vary in capacity due to differences in thickness.

**Figure 12:**
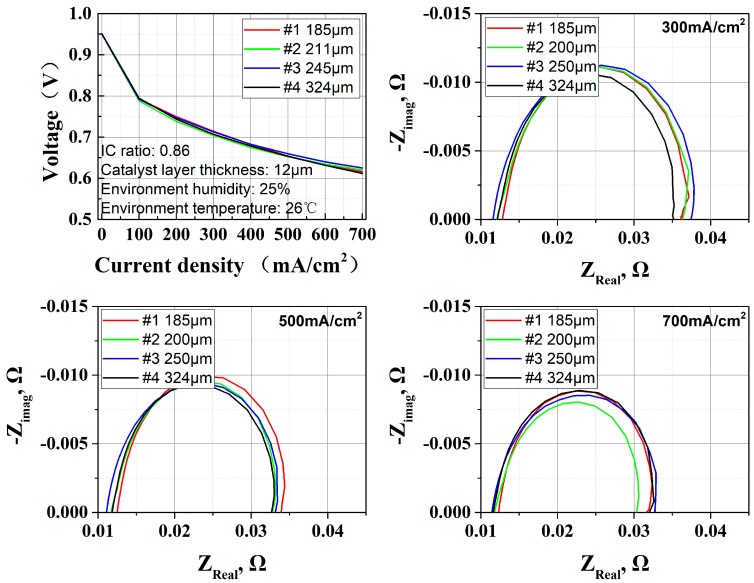
Effect of GDL thickness (185–324 μm) on OC PEMFCs; performance and EIS results show little difference between 4 thickness levels.

The experimental results in Figure 12 show that there is little difference in the performance output of OC PEMFCs equipped with different GDL thicknesses. The water retention capacity is weakly correlated with GDL thickness under forced convection, even though an increase in GDL thickness may prolong the dissipation path of water molecules. Water produced is easily removed by air flow regardless of whether the distance is longer or not. Oxygen transfer and electronic conductivity confirmed this point: according to EIS results, the ohmic resistance and cathode activation resistance did not deteriorate either, within the experimental thickness range (184–324 μm).

**Figure 13:**
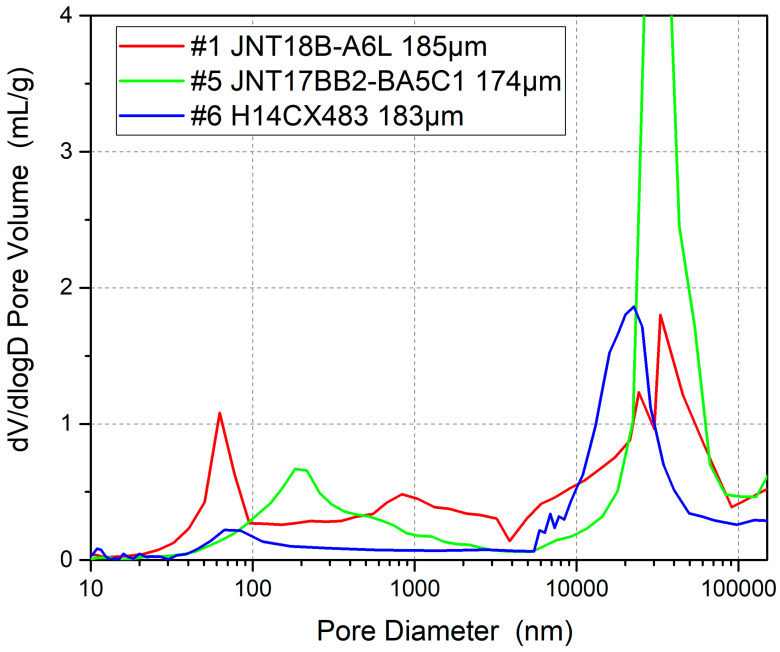
MIP results of #1, #5 and #6 GDLs, which have similar thicknesses, manifesting different pore size distributions.

In addition, GDLs with similar thickness but different pore structures are adopted to investigate the water retention capacity; #1 is medium pore size, #5 is large pore size, and #6 is small pore size in Figure 13.

**Figure 14:**
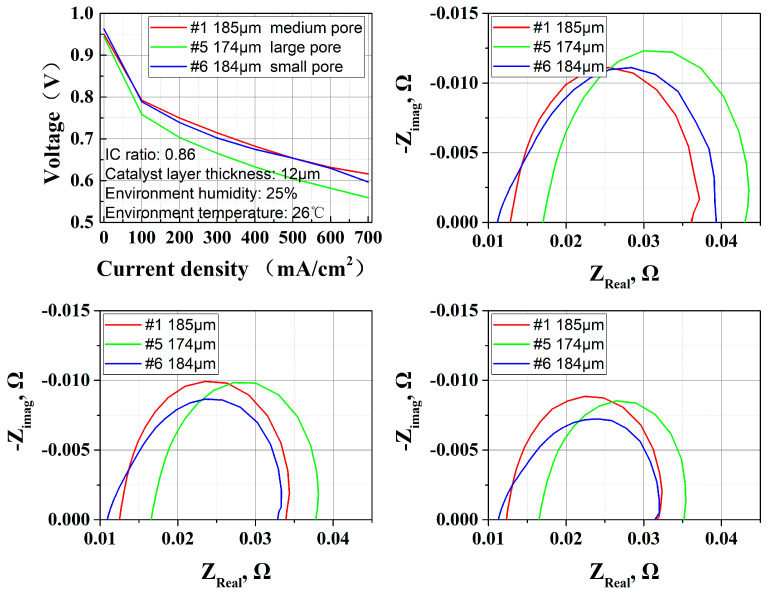
Effect of GDL pore distribution (small, medium and large) with similar thickness on OC PEMFCs; performance and EIS results show that larger pore size leads to 0.005 Ω more ohmic resistance.

Based on experimental results in Figure 14, it was found that the #1 type of GDL had the best performance output with a medium pore size. The #5 GDL had poor performance and its corresponding pore size structure was larger for both MPL and carbon paper substrates. The #6 GDL, which has the smallest pore size distribution among the three samples, exhibits decent performance, but as the current density increases and the stack’s oxygen demand rises, performance degradation occurs due to insufficient oxygen supply.

For OC PEMFCs, the GDL serves as an interface between the CL and the atmospheric environment. On one hand, its excellent air permeability ensures air diffusion into the CL for reaction; on the other hand, its porous structure also acts as a barrier to water escaping from the CL, thereby guaranteeing that sufficient water is retained.

A small pore size enhances capillary force to capture and retain water more efficiently, maintaining the moist state of PEM, reducing the risk of membrane drying, and suppressing water loss in open environments. Under open-cathode conditions, no back pressure was applied, leading to air having more difficulty diffusing through the GDL. A medium pore size strikes a balance between water retention and permeability.

## 4. Conclusions

This study compares the impact of different MEA structures on the performance of OC PEMFCs. By analyzing varying ambient temperatures’ performance and EIS output, we clarify how cathode dehydration adversely affects the ohmic impedance and cathode activation impedance. Furthermore, the influence of cathode CL structures, specifically the I/C ratio and thickness, was investigated as the results shown in Table 5. Results indicate that an I/C ratio around 0.86 delivers optimal performance and enhances resistance to indoor water shortage. A thicker CCL around 12 μm retains moisture more effectively, facilitating additional TPB formation.

The optimization of GDL structures revealed that GDLs with similar pore sizes but varying thicknesses exert minimal influence on performance. In contrast, GDLs with graded carbon paper pore sizes—particularly those with medium-sized pores—significantly improve output.

Through structural optimization, the voltage output of the OC PEMFC increased from 0.563 V@700 mA/cm^2^ to 0.62 V@700 mA/cm^2^. Future studies shall employ the distribution of relaxation times (DRT) method [19] to quantitatively analyze impedance contributions across cell components. Additionally, segment measurement technology [20] can be utilized to evaluate regional power generation efficiency along the planar direction.

## Figures and Tables

**Table 1:** Materials involved in making OC PEMFC MEA.

Materials	Type	Parameter
Catalyst	Umicore 550	platinum content 50%
Solvent	Deionized water, isopropanol, ethanol	purity ≥ 99.7%
Resin	D79	25% resin, 75% water
GDL	#1-JNT18	185 μm
	#2-JNT21	211 μm
	#3-JNT20	245 μm
	#4-JNT30	324 μm
	#5-JNT17	174 μm
	#6-H14CX483	183 μm
PEM	DONGYUE	10 μm
Gasket		45 μm

**Table 2:** Components and parameters involved in OC PEMFC stack.

Item	Type	Parameter
Reaction area		50 cm^2^
Fan	FFB0412SHN	12 V, 0.6 A
Number of cells		3

**Table 3:** Orthogonal experimental design discussing the effect of MEA parameters on performance improvement.

Factor	Level 1	Level 2	Level 3	Level 4
Environmental temperature	20	23	26	/
I/C ratio	0.74	0.8	0.86	0.9
Catalyst thickness	8	12	/	/
GDL thickness	185	211	245	324
GDL pore structure	Small	Medium	Large	/

**Table 4:** GDLs applied for #1–#4, which were selected for GDL thickness effect tests, and #1, #5, and #6 were selected for GDL pore structure tests.

Number	Type	GDL Thickness	Carbon Paper Thickness	MPL Thickness	Pore Structure	Manufacturer
#1	JNT18B-A6L	185 μm	142 μm	43 μm	Medium	JNTG, Hwaseong-si, Republic of Korea
#2	JNT21B-A4G	211 μm	165 μm	46 μm		
#3	JNT20-A6L	245 μm	202 μm	43 μm		
#4	JNT30-A6P	324 μm	280 μm	44 μm		
#5	JNT17BB2-BA5C1	174 μm	143 μm	31 μm	Large	
#6	H14CX483	183 μm	135 μm	48 μm	Small	Freudenberg, Weinheim, Germany

**Table 5:** Orthogonal experimental results.

Factor	Best	Factor Mechanism
Environmental temperature	20 °C	Relatively low temperature reduces water gasification loss
I/C ratio	0.86	Medium I/C can improve proton conductivity while ensuring effective utilization of platinum
Catalyst thickness	12 μm	Increase the pathway for water loss in the catalyst layer
GDL thickness	Insensitive	The thickness has little effect on the water loss under the GDL scale
GDL pore structure	NOT large	The relatively small pore structures help with water retention

## Data Availability

The data is provided upon application, and interested researchers can contact the corresponding author to obtain the data.

## Abbreviations

The following abbreviations are used in this manuscript:

OC PEMFC	Open-cathode proton exchange membrane fuel cells
MEA	Membrane electrode assembly
CL	Catalyst layer
TPBs	Three-phase boundaries
I/C	Ionomer/carbon
EIS	Electrochemical impedance spectroscopy
GDL	Gas diffusion layer
BET	Brunauer–Emmett–Teller
MIP	Mercury intrusion porosimetry
MPL	Microporous layer
